# A peptoid-based inhibitor of protein arginine methyltransferase 1 (PRMT1) induces apoptosis and autophagy in cancer cells

**DOI:** 10.1016/j.jbc.2022.102205

**Published:** 2022-06-25

**Authors:** Mollie A. Brekker, Tala Sartawi, Tina M. Sawatzky, Corey P. Causey, Fatima Khwaja Rehman, Bryan Knuckley

**Affiliations:** 1 Department of Chemistry, University of North Florida, Jacksonville, Florida, USA; 2 Department of Biology, University of North Florida, Jacksonville, Florida, USA

**Keywords:** anticancer drug, cancer, breast cancer, colon cancer, apoptosis, autophagy, peptoid, arginine, methyltransferase, ADMA, asymmetric dimethylarginine, DCM, dichlormethane, DMF, dimethylformamide, HMEC, human mammary epithelial cell, MMA, monomethylarginine, PRMT, protein arginine methyltransferase, SAH, S-adenosylhomocysteine, SDMA, symmetric dimethylarginine, TFA, trifluoroacetic acid

## Abstract

Protein arginine methyltransferases (PRMTs) are *S*-adenosylmethionine-dependent enzymes that transfer a methyl group to arginine residues within proteins, most notably histones. The nine characterized PRMT family members are divided into three types depending on the resulting methylated product: asymmetric dimethylarginine (Type I PRMT), symmetric dimethylarginine (Type II PRMT), or monomethylated arginine (Type III PRMT). In some cancers, the resulting product can lead to either increased or decreased transcription of cancer-related genes, suggesting PRMT family members may be valid therapeutic targets. Traditionally, peptide-based compounds have been employed to target this family of enzymes, which has resulted in multiple tool and lead compounds being developed. However, peptide-based therapeutics suffer from poor stability and short half-lives, as proteases can render them useless by hydrolytic degradation. Conversely, peptoids, which are peptide-mimetics composed of N-substituted glycine monomers, are less susceptible to hydrolysis, resulting in improved stability and longer half-lives. Herein, we report the development of a bioavailable, peptoid-based PRMT1 inhibitor that induces cell death in MDA468 and HCT116 cancer cell lines while not exhibiting any significant impact on nontumorigenic HepaRG or normal human mammary epithelial cells. Furthermore, the inhibitor described herein appears to induce both apoptosis and autophagy, suggesting it may be a less toxic cytostatic agent. In conclusion, we propose this peptoid-based inhibitor has significant anticancer and therapeutic potential by reducing cell viability, growth, and size in breast and colon cancer. Further experimentation will help determine the mechanism of action and downstream effects of this compound.

Arginine methylation is a common posttranslational modification that occurs when methyl groups are transferred to the guanidinyl side chain of arginine residues. The protein arginine methyltransferase (PRMT) family of enzymes are *S*-adenosylmethionine-dependent enzymes that catalyze this transfer, which can result in significant changes to protein–protein interactions, as well as protein–DNA/RNA interactions. Among the most well-studied substrates for PRMT enzymes are the N-terminal tails of histone proteins. Given the critical role that histones play in the packaging of DNA, it is not surprising that posttranslational modifications to the histones affects the translation of some genes (1, 2, 3, 4). Furthermore, dysregulation of these methylating enzymes has been associated with the aberrant expression of some cancer-related proteins, which makes this enzyme family a target for potential therapeutic intervention in cancer treatment (5).

PRMT family members are subcategorized into three types based on their methylation products (Fig. 1). Type I enzymes, a group that includes PRMTs 1, 2, 3, 4, 6, and 8, catalyze the sequential additions of two methyl groups to yield asymmetric dimethylarginine (ADMA) residues. Type II enzymes, which include PRMTs 5 and 9, catalyzed the sequential addition of two methyl groups to yield symmetric dimethylarginine (SDMA) residues. The lone member of the Type III group, PRMT 7, catalyzes the addition of a single methyl group to form monomethylarginine (MMA) residues (6, 7, 8, 9, 10).

**Figure 1:**
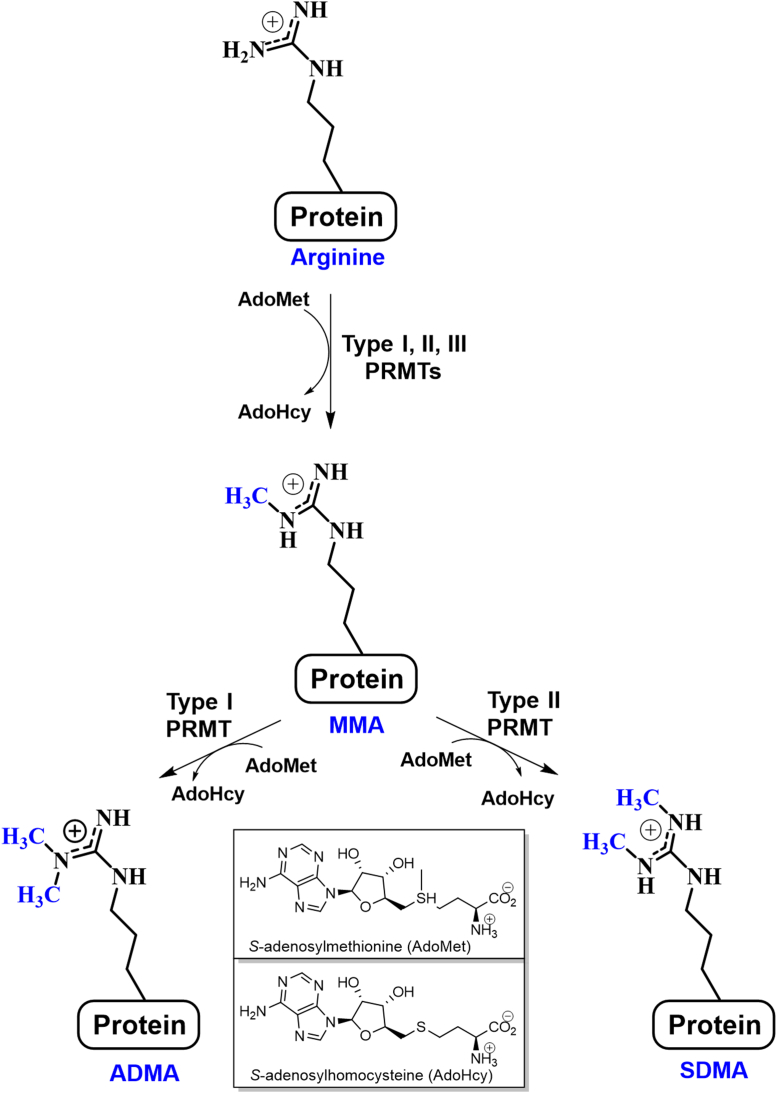
**Protein arginine methyltransferases catalyze the transfer of methyl groups from *S*-adenosylmethionine to the arginine found within proteins.** The family members are categorized into three types: type I (produce asymmetric dimethylarginine; ADMA), type II (produce symmetric dimethylarginine; SDMA), and type III (produce monomethylarginine; MMA).

While there is some overlap in the substrate profiles for type I and II PRMTs, the resulting downstream effects of the methylation products (ADMA *versus* SDMA) can be markedly different. A notable example of differential outcomes due to alternate modification can be found at the R3 residue on the N terminus of histone H4. This arginine is a substrate for both PRMT 1 (type I) and PRMT 5 (type II), thus it can be converted to either ADMA or SDMA, respectively. The conversion of this residue to ADMA is associated with the transcriptional activation of genes under the control of p53 and estrogen receptors, among others (1, 11). Conversely, the conversion of the same residue to SDMA is associated with transcriptional repression of the same genes (1, 12, 13, 14, 15).

More specifically, PRMT1 has been shown to catalyze the production of H4R3me2a, and this specific modification is a key switch of the epithelial–mesenchymal transition at the ZEB1 promoter, activating its transcription leading to breast cancer (16). Further studies have also identified overexpression of PRMT1 in some cancers, including leukemia, prostate, esophageal, lung, bladder, and breast compared to normal cells leading to increased protein levels and promoting cell proliferation (5, 17, 18, 19). These and other studies have identified PRMTs, specifically PRMT1, as a key contributor to the proliferation of cancers through various mechanisms including epigenetic-mediated gene expression (20, 21, 22, 23).

To date, several PRMT inhibitors have been reported, including some that are isozyme specific. One of the first non-AdoMet–based pan inhibitors for Type I PRMTs was developed in 2004 but only recently has a PRMT inhibitor made it to clinical trials (24, 25). Given that PRMTs modify protein substrates, it is not surprising that many peptide-based compounds have been developed to study these enzymes (26, 27, 28). One obvious advantage of peptide-based inhibitors is the modularity and the natural recognition elements conveyed by the amino acid sequence, which can be used tune substrate specificities. For example, we recently reported the development a peptide-based chemical probe that exhibits selectively for PRMT1 over PRMT 5 *in vitro* (29). One major limitation of peptide-based compounds as *in vivo* tools and therapeutics is their inherent susceptibility degradation by proteolysis.

Recently, we began exploring the use of peptoid scaffolds as an alternative to peptides. Peptoids, which are polymers of N-substituted glycine residues, piqued our interests as a potential surrogate for traditional peptide backbones because as tertiary amides, they are less susceptible to hydrolytic degradation. However, these structures still allow a level of modularity that is comparable to peptides and can be constructed rapidly by submonomer synthesis (30, 31). Like peptides, specific sequence design may provide selectivity among various enzymes, improving the *in vivo* utility and making them suitable clinical therapeutics. Peptoid-based inhibitors have been previously developed for enzymes and used as antimicrobial compounds, further demonstrating their efficacy in drug discovery (32, 33, 34, 35, 36). Initial studies found that peptoid-based analogs of H4-16, a known PRMT substrate, were not modified by the PRMT enzymes; instead, these analogs were found to inhibit the enzyme at low millimolar concentrations (37). This finding led us to pursue the development of peptoid-based compounds as selective inhibitors of PRMTs.

Herein, we describe the development of a peptoid-based inhibitor that selectively inhibits PRMT1 over PRMT5 with an IC_50_ value in the low micromolar range. Additional *in vitro* studies have shown that this compound significantly reduces cell viability and growth potential in MDA468 breast carcinoma and HCT116 +/+ colon carcinoma cell lines, with no noticeable toxicity in normal and nontumorigenic cells. Further analysis reveals this inhibitor induces apoptosis and autophagy to stimulate growth arrest and cell death in cancer cells.

## Results

### Synthesis of warhead peptoids

**Figure 2:**
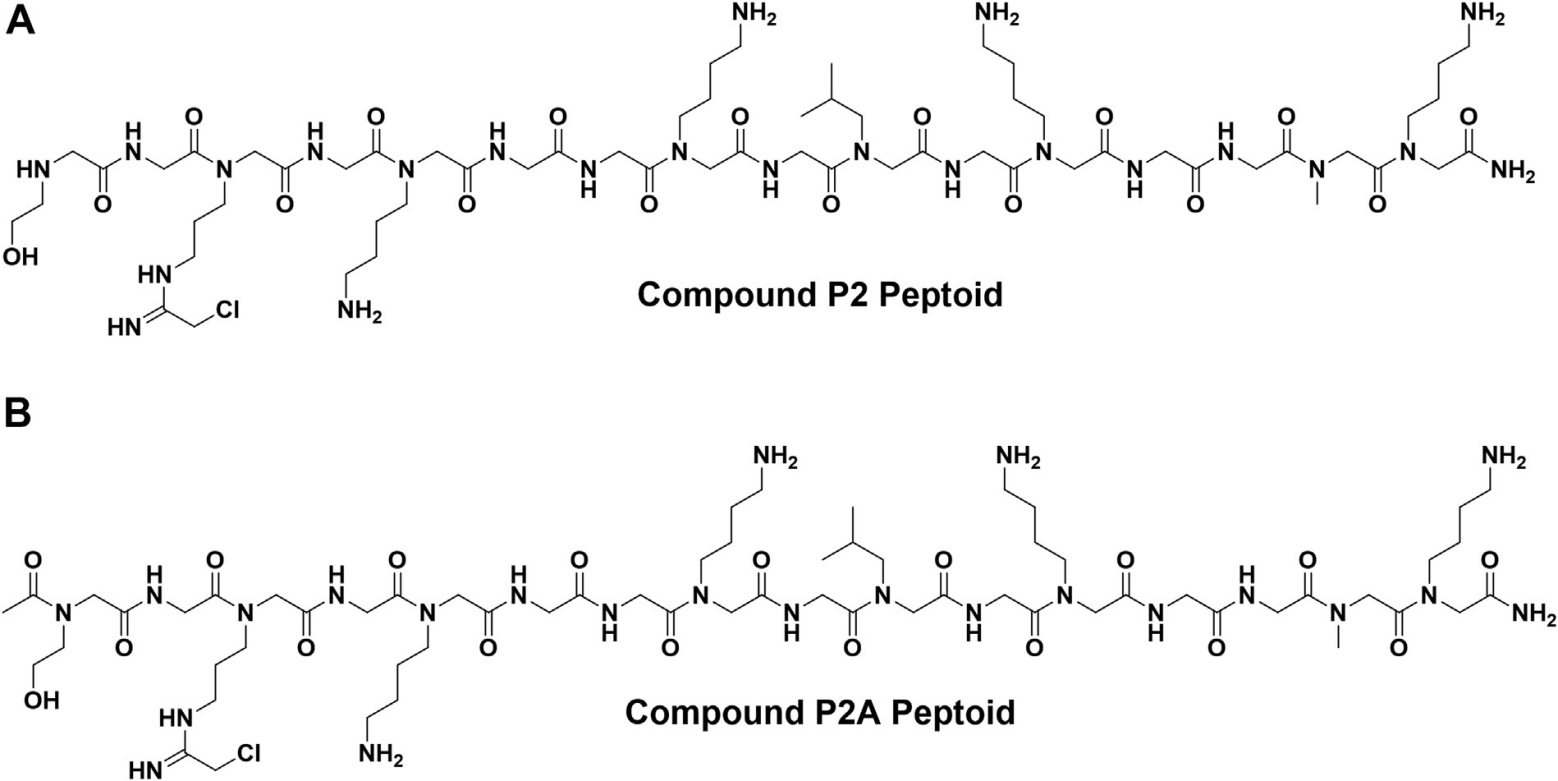
**Peptoid incorporating the chloroacetamidine warhead.***A*, compound P2, unacetylated N-terminus; *B*, compound P2A, acetylated N-terminus.

Histone H4-16 peptoids have been shown to be moderate inhibitors of PRMT1 with some degree of selectivity over PRMT5. In addition, this peptoid contains only a single N-Arg residue, H4R3, in its sequence, which simplifies the analysis and can be used for the development of second-generation histone-based peptoid inhibitors. Modifying this single N-Arg residue to incorporate a reactive warhead would provide the ability to bind to the PRMT1 active site and potentially lead to greater inhibition and selectivity. PRMT1 contains a cysteine (Cys 101) within the active site, but this residue is not conserved in most other PRMT isozymes. Replacing H4R3 with a chloracetamidine warhead, a well-characterized Cys modifier, could lead to selective, irreversible modification of PRMT1. For this reason, we chose to construct an H4-16 peptoid replacing Arg3 with a chloracetamidine warhead (Fig. 2). To determine if acetylation was important in regard to these peptoids, we synthesized both an unacetylated (compound P2) and acetylated version (compound P2A) of the H4-16 warhead peptoid. Compounds P2 and P2A were synthesized and purified, then verified by ESI-MS with a mass of 1460.8 and 1502.8, respectively (Table S1).

### Determination of IC_50_ values for compound P2 and P2A

**Figure 3:**
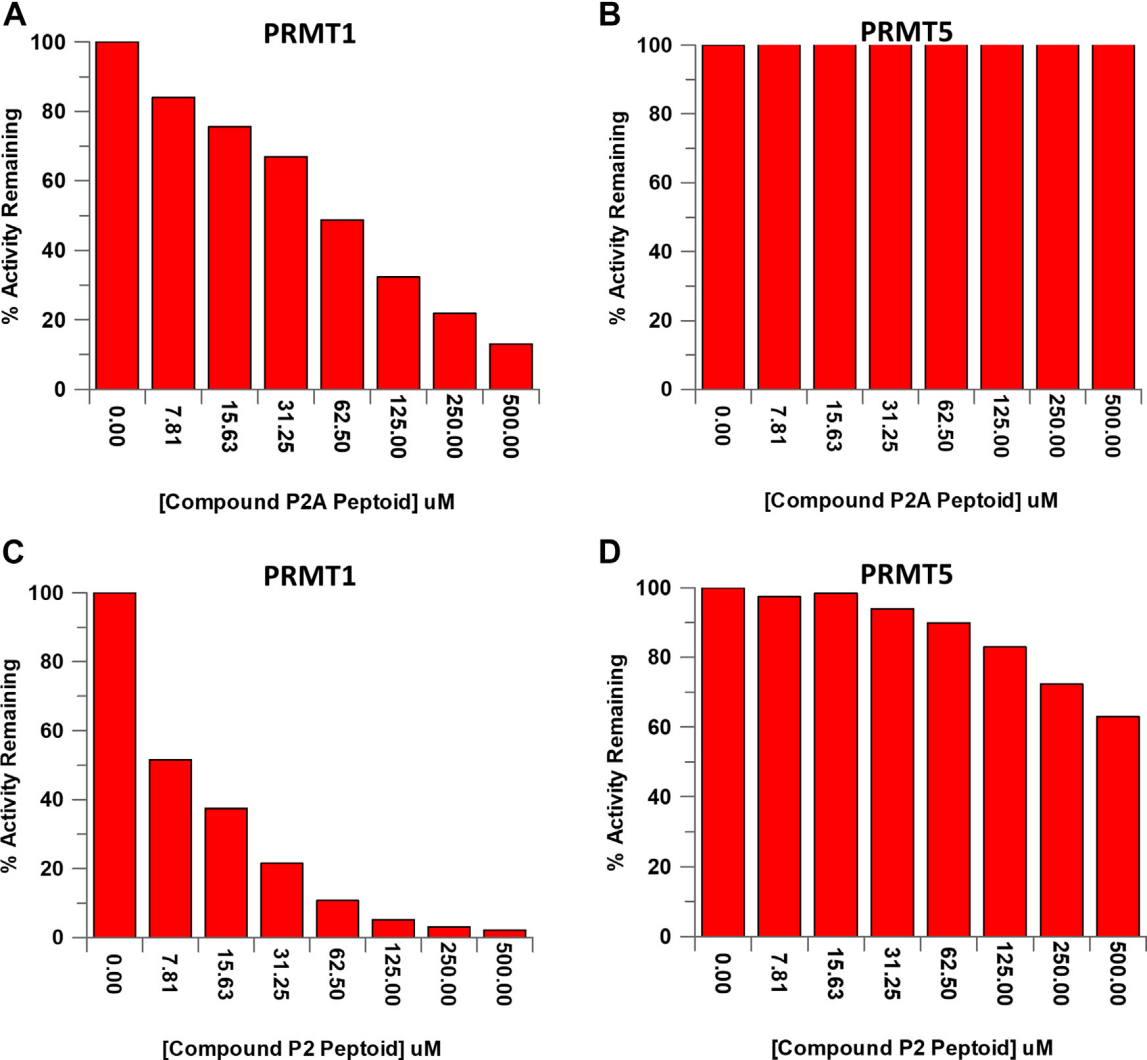
**Compound P2 inhibits PRMT1 at low μM concentrations and exhibits selectivity over PRMT5.** IC_50_ plots of compound P2A peptoid with (*A*) PRMT1 and (*B*) PRMT5. IC_50_ plots of the compound P2 peptoid with (*C*) PRMT1 and (*D*) PRMT5. PRMT, protein arginine methyltransferase.

Compounds P2A and P2 were expected to have improved potency and selectivity as compared to the H4-16 peptoids that lacked the ability to irreversibly modify PRMT1 (37). To this end, we conducted an IC_50_ analysis of these compounds for PRMT1 using a standard methyltransferase assay, in addition to evaluating their ability to selectively target PRMT1 over PRMT5. PRMT1 was treated with compound P2A and resulted in an IC_50_ value of 59.1 ± 2.21 μM (Fig. 3*A*), which is improved potency over the original AcH4-16 peptoid (15-fold; 916 ± 19.5 μM). However, compound P2 resulted in a IC_50_ value of 8.73 ± 0.314 μM with respect to PRMT1 (Fig. 3*C*) and a 45-fold increase in potency compared to the original unacetylated H4-16 peptoid (IC_50_ value for PRMT1 of 396 ± 31.4 μM). These data support previous studies that a positively charged N terminus leads to increased binding and inhibition of PRMT1 (38). Furthermore, we sought to determine if the Cl-warhead, which selectively modifies Cys residues, would provide further selectivity for PRMT1 over PRMT5. The IC_50_ value for compounds P2 and P2A warhead peptoids with PRMT5 were both determined to be >500 μM (Fig. 3, *B* and *D*). Compound P2 exhibited >60-fold difference in IC_50_ values and demonstrated improved selectivity for PRMT1 over PRMT5. The difference in IC_50_ values between PRMT1 and PRMT5 was not surprising but provides evidence that we can selectively target individual members of this family to develop isozyme-specific, peptoid-based inhibitors. Given the improved inhibition and selectivity, compound P2 was used for further studies.

### Effects of compound P2 on cell viability

**Figure 4:**
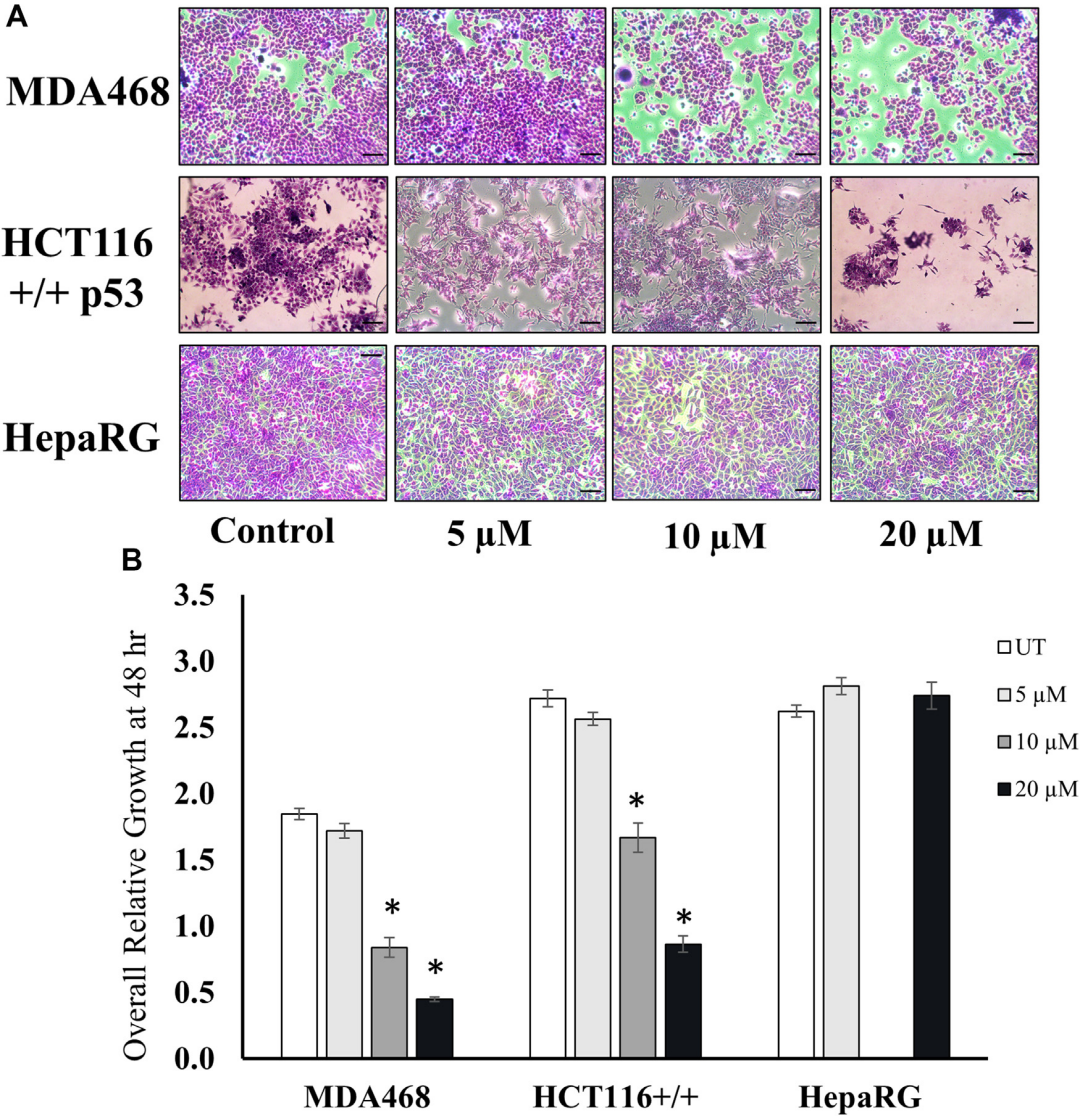
**PRMT inhibitor reduces cell growth and viability specifically in cancer and not in normal cells.***A*, images form the crystal violet assay displaying cell viability in MDA468, HCT116 +/+ p53, and HepaRG cells after 48 h treatment with P2. The scale is 100 μm. *B*, overall relative growth quantification after 48 h in the presence and absence of compound P2. ∗marks significance *p* < 0.01 compared to untreated control for each cell line. Error bars represent standard error from all replicates of the experiment. Each condition was done in triplicate, and the entire experiment was repeated twice for n = 6 samples per group. PRMT, protein arginine methyltransferase.

To study the anticancer potential of compound P2, MDA468 breast carcinoma and HCT116 colon carcinoma cells along with HepaRG immortalized, terminally differentiated liver cells were grown in the presence of various concentrations of compound P2 (0–20 μM) for 48 h, and cell viability was assessed using the crystal violet assay. Compound P2 had significant antiproliferative effect in a dose-responsive manner on both cancer cell lines, while no prominent change was seen in the viability of the nontumorigenic HepaRG cells (*p* < 0.01) (Fig. 4, *A* and *B*). The proliferation was reduced most significantly in MDA468 cells (55% at 10 μM and 75% at 20 μM). Although HCT116 responded to the compound well, the effects were less pronounced with 40% reduction at 10 μM and 60% at the highest concentration (Fig. 4*B*).

**Figure 5:**
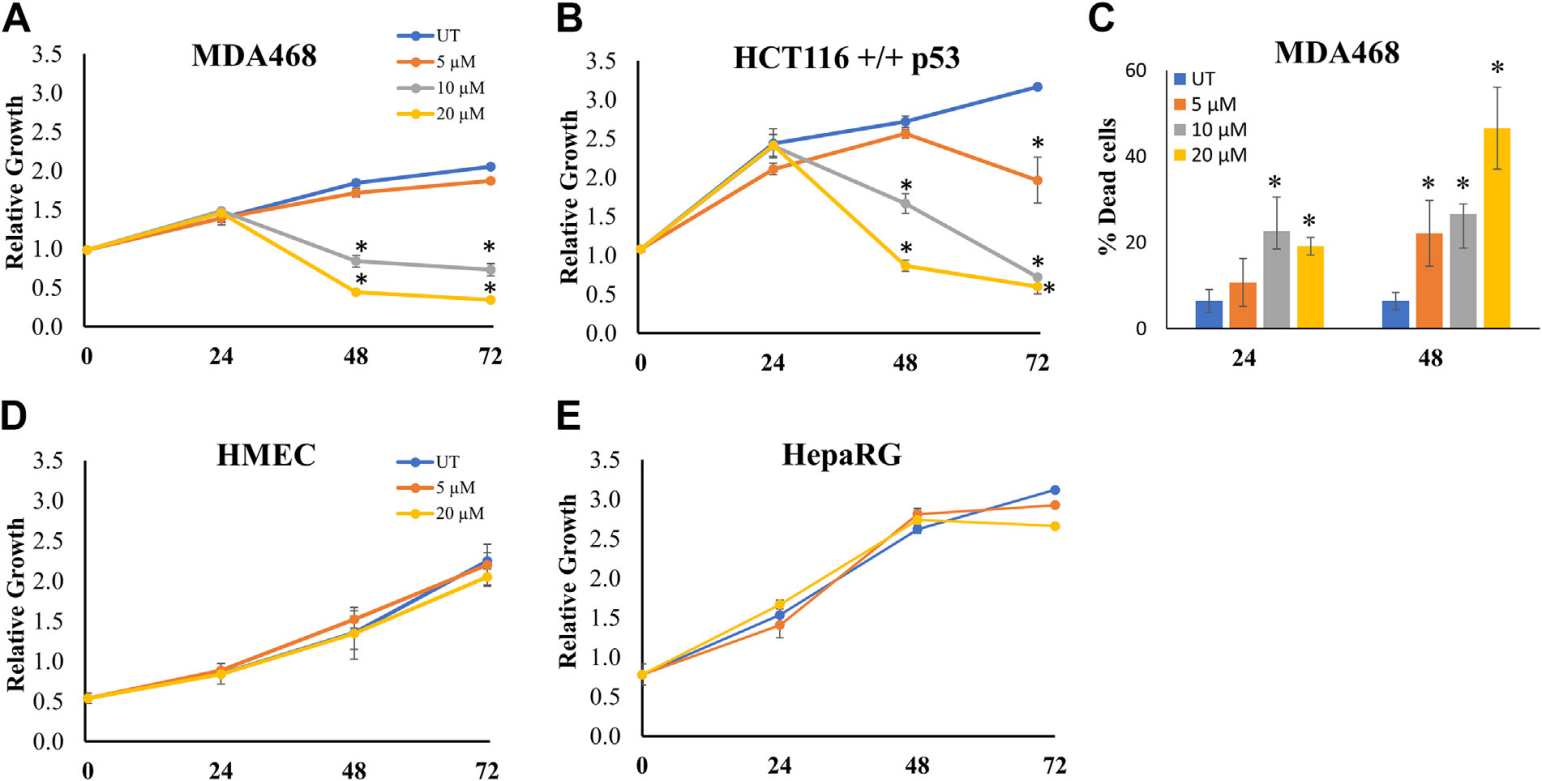
**PRMT inhibitor reduces viability of cancer cells in a dose- and time-dependent manner.** Relative growth was observed and measured in (*A*) MDA468, (*B*) HCT116 +/+ p53, (*D*) HMEC, and (*E*) HepaRG cell lines over 72 h of P2 treatment. Each treatment was done in duplicate, and the entire experiment was repeated thrice for n = 6/group. *C*, total number of dead cells for MDA468 cells were measured using the trypan blue exclusion assay. Each condition was done in duplicate, and the entire experiment was repeated twice. ∗ marks significance *p* < 0.01 compared to untreated control for each cell line. Error bars represent standard error from all replicates of the experiment. HMEC, human mammary epithelial cell; PRMT, protein arginine methyltransferase.

Next, we examined the specificity of compound P2 on cancer cells over 72 h time course using a panel of cancer and normal cells in the crystal violet assay. Once again, there was a dose- and time-dependent decrease in cell viability in both cancer cell lines (Fig. 5, *A* and *B*). In addition, trypan blue exclusion analysis showed significant increase in dead and dying cells at both 24 h and 48 h posttreatment with P2 in a dose-response manner in both MDA468 and HCT116 cancer cells (Fig. 5*C* and data not shown). In contrast, nontumorigenic HepaRG and normal human mammary epithelial cells (HMECs) showed no detectable effect in growth (Fig. 5, *D* and *E*). These results suggest that compound P2 specifically targets cancer cells and reduces their viability and growth potential without significant toxicity to normal and nontumorigenic cells.

**Figure 6:**
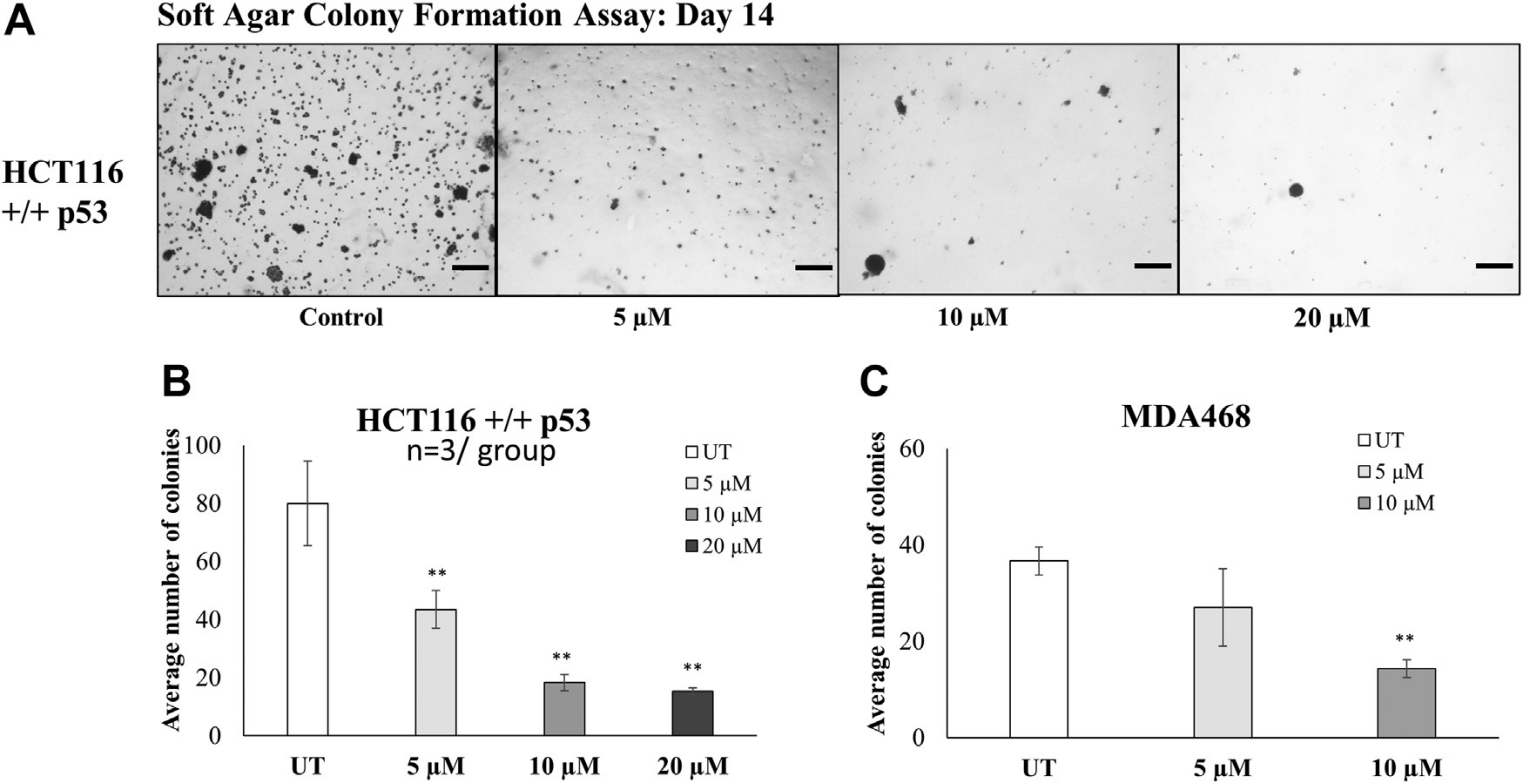
**PRMT inhibitor reduces tumorigenicity of cancer cells.***A*, the colony formation assay measured the cell’s ability to form and maintain colonies over 14 days. The scale is 100 μm. Treatment with compound P2 reduces number of colonies in a dose-dependent manner in both (*B*) HCT116 +/+ p53 and (*C*) MDA468 cells. ∗∗ marks significance *p* < 0.01 compared to untreated control for each cell line. Error bars represent standard error from all replicates of the experiment. Each condition was repeated in three separate wells, and three fields/well were photographed for quantification and analysis.

After studying the effect of P2 treatment on the viability and growth of cells, the impact of the drug was tested on the cells ability to form and maintain colonies over 14 days (Fig. 6, *A*–*C*). The colony formation assay results show a significant dose-dependent decrease in the number of MDA468 and HCT116+/+ colonies formed posttreatment with P2. A decrease of approximately 50% in the number of HCT116 colonies was seen with 5 μM P2 (Fig. 6*B*) and with 10 μM P2 treatment in MDA468 (Fig. 6*C*). These results confirmed the ability of P2 to reduce tumorigenicity of cancer cells.

### Compound P2 induces apoptosis and autophagy in cancer cells

**Figure 7:**
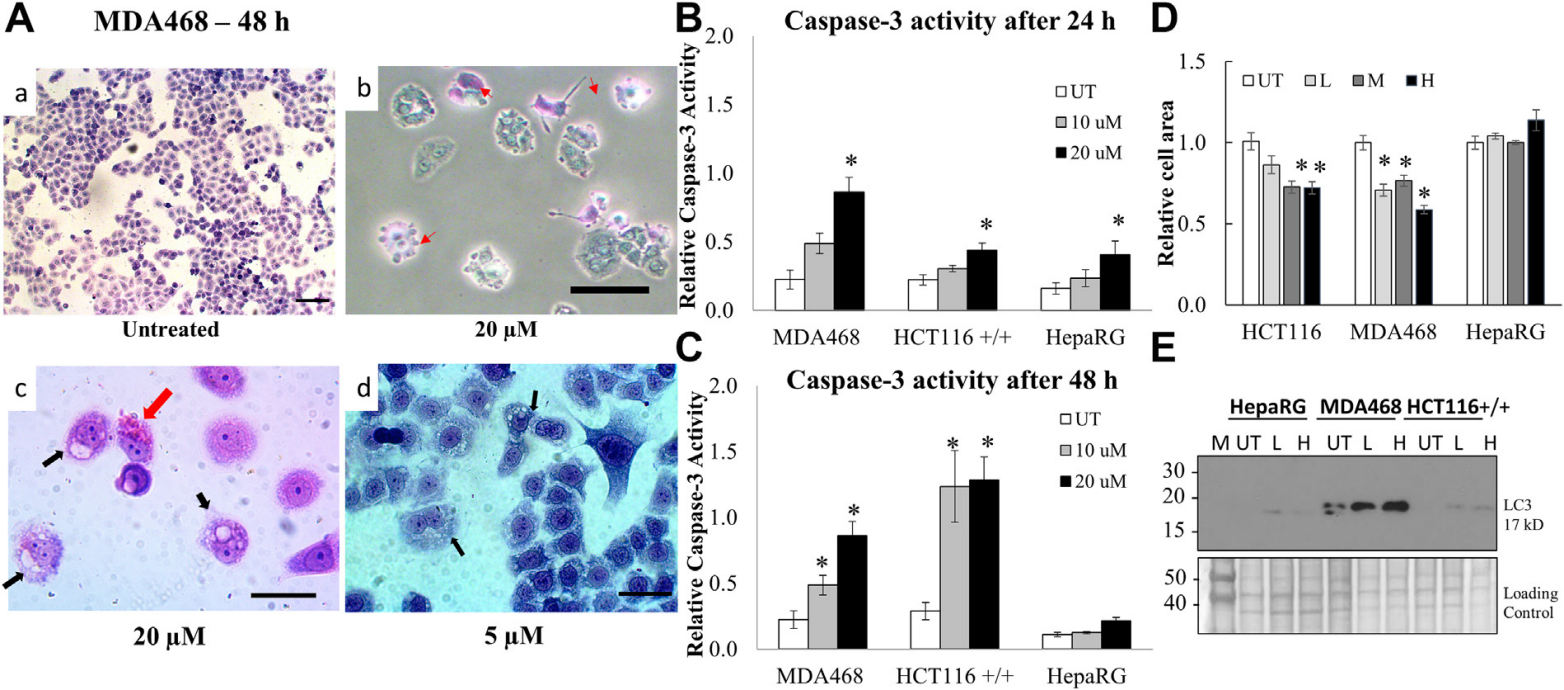
**P2-treated cancer cells show evidence of both apoptosis and autophagy *in vitro*.***A*, MDA468 cells treated with compound P2 show membrane blebbing (*red arrows*) and the appearance of small vacuoles (*black arrows*) suggesting both apoptosis and autophagy are occurring. a and b are 100 μm scale, and c and d are 40 μm scale. Caspase-3 activity was measured in MDA468, HCT116 +/+ p53, and HepaRG cells over (*B*) 24 h and (*C*) 48 h. *D*, relative cell area in untreated (UT), low (L; 5 μM), medium (M; 10 μM), and high (H; 20 μM) concentrations of compound P2. ∗ marks significance *p* < 0.01 compared to untreated control for each cell line. Error bars represent standard error from all replicates of the experiment. *E*, the presence of LC3 in response to compound P2 was measured by Western blot analysis in all three cell lines. The molecular weight ladder is labeled M.

To examine the mechanism of anticancer activity, we next observed cells treated with compound P2 after staining for morphological changes in response to the drug. Untreated cells remained healthy and normal in appearance throughout the experimental time frame, while several cells in the treated samples showed membrane blebbing indicative of apoptosis and presence of small vacuoles in rounded cells suggestive of autophagy (Fig. 7*A*). To test for apoptosis, caspase-3 activity was examined in protein extracts from untreated and treated cells. Significant caspase-3 activity was seen in a dose and time-response manner in MDA468 and HCT116 cells after 24 h and 48 h of treatment with compound P2 (Fig. 7, *B* and *C*; *p* < 0.02). In contrast, minimal activation of caspase-3 was seen in HepaRG cells and only at the highest dose at 24 h (Fig. 7, *B* and *C*). Combined, these results indicate apoptosis as a mechanism of action for the observed anticancer activity of compound P2.

In addition, we examined if autophagy was also contributing to the reduced viability seen after treatment with compound P2 as a large percentage of treated cells showed presence of vacuoles (Fig. 7*A*). First, we measured cell areas using ImageJ analysis in treated and untreated stained cells grown on coverslips as a common morphological change in cells undergoing autophagy is a reduction in size. Results showed significant decrease in size of cells in both cancer cell lines post compound P2 treatment, while HepaRG remained unchanged (Fig. 7*D*). This reduction in cell size could also be an additional sign of active autophagy (Fig. 7*D*). To confirm, we monitored the levels of microtubule-associated protein light chain 3 (LC3), a well-known marker of autophagy, using Western blot analyses in all three cell lines as a response to compound P2 treatment. The data support active autophagy though the induction was much less pronounced in the nontumorigenic HepaRG (Fig. 7*E*). Taken together, the results find both apoptosis and autophagy are important pathways activated by compound P2 to induce growth arrest and cell death in cancer cells.

## Discussion

PRMT family members catalyze the methylation of arginine residues in a variety of proteins but have been extensively studied in relation to their modification of the N-terminal tails of histone proteins. PRMT isozymes have varying substrate specificity in regard to these histone tails. For example, PRMT1 and PRMT5 are primarily responsible for more than 90% of the methylation of Arg3 on histone H4. Meanwhile, PRMT4 (CARM1) does not methylate H4R3 but is responsible for the majority of methylation found on Arg17 on histone H3. These similarities and differences in substrate specificity provide an opportunity to design inhibitors targeting either single PRMTs or multiple family members. Peptides based on the N-terminal tail of these histones have been shown to have kinetic parameters, *k*_*cat*_ and *K*_*m*_, that are similar to those of native histones. Peptides present a unique niche of pharmaceutical compounds that provide distinct physical and biochemical features to specifically bind to disease-related proteins. For example, peptides have been used as synthetic signaling molecules to regulate physiological functions and have also been used to disrupt protein–protein interactions (39, 40). Furthermore, the ease of synthesis and low cost of production have made peptides favorable compounds as disease therapeutics. However, one of the main challenges in developing peptide-based therapeutics is their poor stability and short half-lives due to proteolytic degradation. The use of peptoids, which are peptide-mimics displaying the amino acid side chain on the nitrogen of the amide backbone instead of the α-C, mitigates the problem of proteolysis. Furthermore, these compounds generally display greater cellular permeability than their peptide analogs, which also improves their potential as pharmaceuticals (41, 42).

As a result, peptoid versions of the histone H4 N-terminal tail (H4-16) were recently developed by replacing the amino acids residues with peptoid monomers, thus shifting the side chain from the α-C to the nitrogen on the backbone. However, the peptoid version of H4-16 was a very poor PRMT1 substrate with *k*_*cat*_*/K*_*m*_ values ∼10^−17^ M^−1^ min^−1^. Interestingly, the peptoid version was a moderate inhibitor of PRMT1 with IC_50_ values in the high micromolar range (∼400–900 μM). Although the values are fairly high for pharmaceutical development, they did provide a novel scaffold for inhibitor design targeting the PRMT family (37). Thus, this scaffold was used to develop compounds that have enhanced binding and specifically target PRMT1.

Based on this information, we replaced the N-Arg side chain of the peptoid with a chloracetamidine warhead to develop a more potent and selective compound. The chloracetamidine ‘warhead’ in place of the guanidinium moiety of the arginine side chain, specifically Arg3 on the histone H4 N-terminal tail, allows for specific PRMTs to be covalently modified by this compound through inactivation of PRMT1 by formation of a thioether adduct with Cys101 (43). This strategy was previously used to develop some peptide-based tool compounds against PRMT1, however given the advantages of peptoids over peptides, we incorporated this chloracetamidine warhead into the H4-16 peptoid. Based on the IC_50_ values, the incorporation of the warhead into the acetylated H4-16 peptoid (compound P2A) increased the potency more than 15-fold as compared to the nonwarhead version. Moreover, replacing NArg3 with the warhead in the unacetylated H4-16 peptoid (compound P2) resulted in >45-fold increase in potency. As PRMT1 and PRMT5 are responsible for more than 90% of all protein arginine methylation in mammals, we measured the low micromolar inhibitors against PRMT5 to determine selectivity between these two isozymes. Based on the IC_50_ values, compound P2 is selective for PRMT1 over PRMT5 by more than 60-fold, based on the IC_50_ values. The warhead-based peptoids result in improved potency, selectivity, bioavailability, and half-life potential as compared to the peptide versions. However, the peptide versions of these compounds were also synthesized, and the IC_50_ values were measured to compare the role that the peptoid backbone may play in contributing to inhibition (Fig. S1). The AcH4-16 warhead peptide had an IC_50_ value similar to the peptoid version (46.9 ± 4.86 μM compared to 59.1 ± 2.21 μM). Whereas, the unacetylated H4-16 warhead peptoid (compound P2) was ∼3-fold better than the peptide version (29.6 ± 5.16 μM *versus* 8.73 ± 0.314 μM; Fig. S2). In total, this suggests the peptoid-based inhibitors improve potency and possess better stability overall, while also improving selectivity over the peptide version as compared with PRMT5.

Given the role of PRMTs in cancer, such as breast and colon cancer, we evaluated the bioactivity of compound P2 in MDA468 and HCT116 cell lines compared to both HepaRG and HMECs. Compound P2 showed antiproliferation activity in a dose-dependent manner in both tested cancer cell lines, MDA468 and HCT116+/+, without interfering with the growth of normal cell lines HMEC and HepaRG. This suggests that compound P2 has both specificity and anticancer activity. This inhibitory effect is consistent with the result seen in experiments studying PRMT pan inhibitors AMI-1 and S-adenosylhomocysteine (SAH) conducted by Janisiak *et al.* (44) where they observed a dose-dependent decrease in cell viability of Rh30 and RD Rhabdomyosarcoma cell lines with the administration of the inhibitors.

Given that AMI-1 and SAH are pan PRMT inhibitors, inhibition of PRMT1 was seen by a reduction in the levels of PRMT1 methylated product, H4R3me2a. Likewise, Eram *et al.* (45) also saw similar results of decreased viability using MS023, a PRMT type I pan inhibitor. MS023 showed a dose-dependent reduction in cell viability and a downstream inhibition of both PRMT1 and PRMT6 by the undermethylation of H4R3 and H3R2, respectively (37). Unlike the mentioned PRMT inhibitors, compound P2 is a PRMT1-specific peptoid inhibitor, and the chlorine warhead brings this specificity to the molecule. However, the *in vitro* specificity of compound P2 against PRMT1 was not confirmed in this study.

Compound P2 was effective in both tested cancer cell lines, but MDA468 cells showed less but more sustained response while HCT116 had a delayed, but more effective response. This difference is potentially explained by the unique genetic makeup of each cell line, the different pathways activated, and the protein profile of each, with MDA468 lacking both the methylthioadenosine phosphorylase (*MTAP*) and *p53* genes. Fedoriw *et al.* (46) looked at type I PRMT inhibitor, GSK3368715, across 249 cell lines and 12 tumor types, each showing a decrease in proliferation to a different extent. These results suggest that each cell line has its own IC_50_ value. In addition, they suggested that the loss of the *MTAP* gene may increase the susceptibility of cell lines to PRMT type I inhibitors through synergistic inhibition of PRMT5 due to the accumulation of 2-methylthioadenosine, a natural PRMT 5 inhibitor. MTAP is a tumor suppressor gene near *CDKN2A* and can be co-deleted in cancer cell lines. This proposed a possible explanation for early sensitivity in MDA-MB-468 to compound P2 during the time course experiments. However, as MDA468 has a mutated *p53*, this lack of fully functional *p53* may contribute to the limited efficacy of P2.

The increased caspase-3 activity in both cancer cell lines compared to the normal cells suggests a role for a tumor-specific apoptosis mechanism of action by compound P2. This was supported by the observance of apoptotic vacuoles in cells treated with the inhibitor. Apoptosis has been seen as the common cause of cell death after treatment with PRMT pan inhibitors due to the downregulation of cyclin D. Cells treated with PRMT inhibitors also exhibit cell cycle arrest at the G1/S (47, 48). Cell cycle studies looking at HepaRG-FUCCI cells suggest that treatment with compound P2 inhibited the cells from re-entering the cell cycle. Western blot data looking at the expression of cyclin D in the three cell lines, MDA468, HCT116, and HepaRG, suggest a downregulation in cyclin D expression posttreatment (data not shown). However, the significance of this downregulation is yet to be verified.

**Figure 8:**
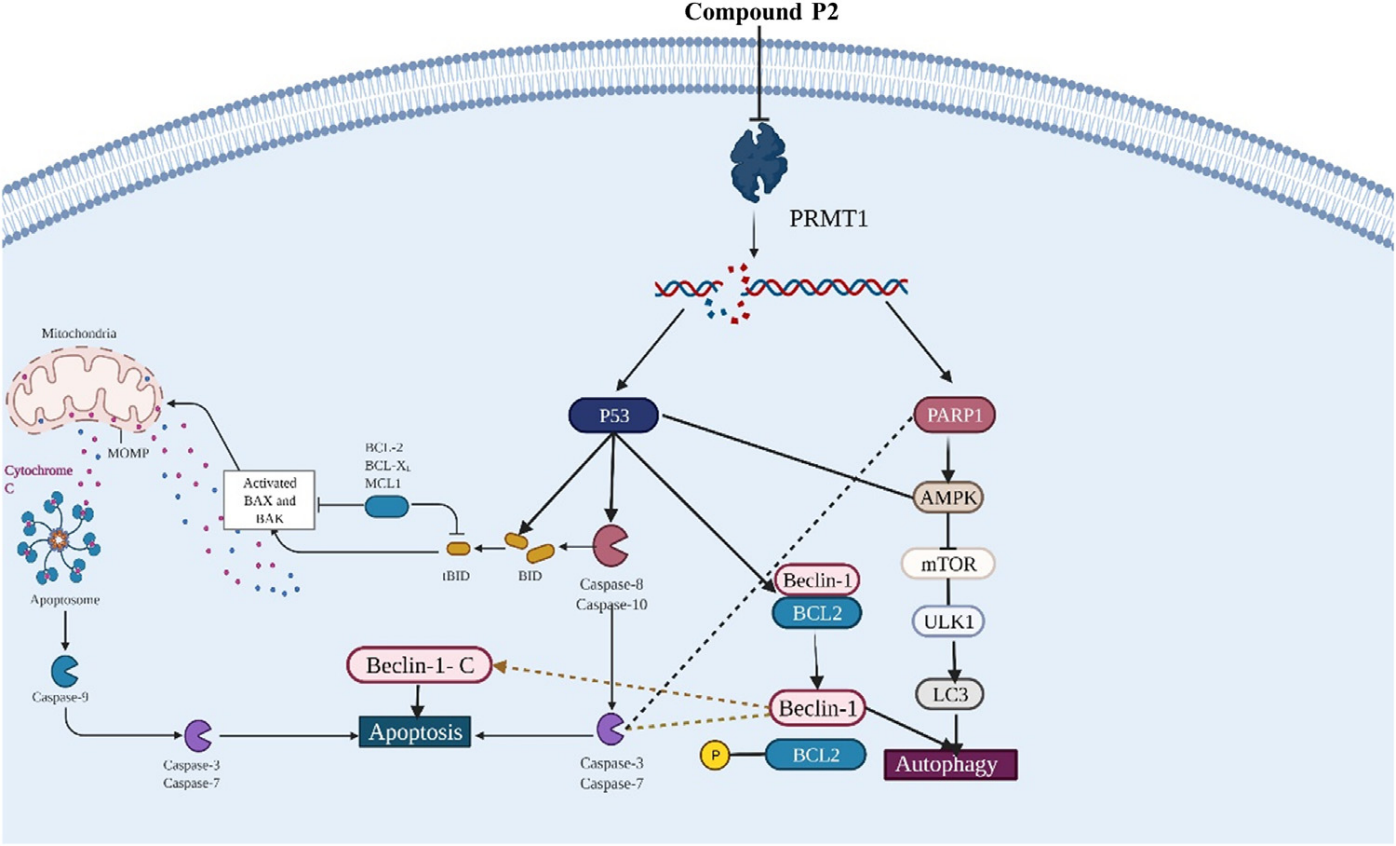
**Suggested mechanism of action resulting from treatment with compound P2 leading to autophagy and apoptosis in MDA468 and HCT116 +/+ p53 cell lines**.

The morphological studies show the presence of multiple vacuoles and the reduction in the average size specifically in cancer cells treated with compound P2, and the increased expression of LC3 in all three cell lines. Western blots suggest that autophagy may also play a role in the mechanism of action of compound P2. Autophagy is often the go-to survival response of the cell after it undergoes stress or starvation. It is seen as a protective method utilized before cell suicide *via* apoptosis. Caspases are a primary contributor to apoptotic cell death, but recent studies have also shown they can directly interact with core autophagy proteins, slowing down the autophagic process to preserve survival. Under most conditions, caspases inhibit autophagy and induce apoptosis. However, caspase-1, -3, -8, and -9 have favored an autophagy response (48). It may be possible that Caspase-3 levels are increased to quench autophagy, as many a-ketoglutarates have been discovered as substrates for caspases. These previous studies suggest a link between apoptosis and autophagy because of caspase activity. Thus, compound P2 may elicit a similar response in these cells, leading to growth arrest and cell death. In their review, Tsapras and Nezis (48) suggest that specific caspases, including caspase-2, may promote autophagy in the presence of mitochondrial oxidation damage. Prolonged exposure to compound P2 would likely push the cell from undergoing autophagy into apoptosis (Fig. 8).

## Conclusion

The peptoid-based inhibitor, compound P2, has a greater potential over peptide-based compounds for development into therapeutics against PRMT-associate diseases. Overall, this PRMT inhibitor shows promising anticancer activity in MDA468 and HCT116 cell lines, reducing cell viability, relative growth, and cell size. Further experimentation regarding its specificity, bioavailability, synergistic ability, and its mechanism of action are necessary. Conducting experiments to confirm the specificity of compound P2 against PRMT1 and to assess the bioavailability and half-life of the peptoids *in vitro* is essential for determining its efficiency as a drug. The selectivity of compound P2 can be confirmed by running Western blots looking at the levels of PRMT1 and its products (*e.g.*, H4R3me2a) in treated *versus* controlled cell lines. These data would help establish a more precise understanding of the downstream effects of this compound. Another helpful comparison would be between cells treated with compound P2 and a cell line with PRMT1 knockdown, *e.g.*, MDA-MB-231. These studies, and others, will be conducted in the future in addition to elucidating the mechanism of action by compound P2 while also helping to shed light on a possible mechanistic pathway of the drug and the genes being transcribed posttreatment.

## Experimental procedures

### Materials

The peptoid and peptide synthesis reagents, including bromoacetic acid, diisopropylcarbodiimide, *N*-methylmorophiline, methylamine, isobutylamine, and piperidine were purchased from Sigma Aldrich. Solvents and various other reagents including triethylamine, acetic anhydride, dimethylformamide (DMF), trifluoroacetic acid (TFA), triispropylsilane, diethylether, and standard biochemical reagents were purchased from Fisher Scientific. Fmoc-Gly-OH, hexafluorophosphate benzotriazole tetramethyl uronium, dichlormethane (DCM), and Fmoc-MBHA rink amide resin were purchased from VWR. The MTase-Glo methyltransferase assay kit was purchased from Promega for completing kinetic analyses. PRMT1 and PRMT5 were expressed and purified as previously described (49).

### Antibodies and reagents

Protease inhibitor cocktail (P8340), phosphatase inhibitor cocktail 2 (P5726) and 3 (P0044), and dimethyl sulfoxide (D8418) were purchased from Sigma-Aldrich. The primary antibodies used in this study from Santa Cruz biotechnology were cyclin D1 (sc-753); cyclin A (sc-596); cyclin B1 (sc-245). Additionally, we used pHH3ser10 (9701A; Cell signaling), LC-3 (L8918; Sigma), cleaved caspase-3, and PARP (cell signaling) and GAPDH (IMG-5019A-2; IMGENEX). Horseradish peroxidase-conjugated secondary antibodies of goat anti-rabbit (4010-05) and goat anti-mouse (1012-05) were purchased from Southern Biotech.

### Peptide synthesis

The peptides incorporating the chloracetamidine warhead in place of the Arg residue in histone H4-16 were synthesized according to previously described methods (29). The mass of peptides was confirmed by electrospray ionization mass spectrometry (ESI-MS; Table S1).

### Peptoid synthesis

The synthesis of peptoids was completed on Fmoc-MBHA rink amide resin (0.55 mmol/g). The resin was treated with 20% piperidine in DMF (5 ml for 15 min, twice) to remove the Fmoc-protecting group. Individual peptoid monomers were added by first incubating the resin with a 1:1 mixture of 1 M bromoacetic acid and 1 M diisopropylcarbodiimide in DMF (2 ml) and microwaved for 30 s total (6 × 5 s at 100% power; venting and rocking for 30 s in between intervals). High purity (>99.8%) DMF is required for the coupling steps. The resin was washed with DMF (5 × 5 ml) to remove any residual reagents. The resin was then treated with a 0.5 M solution of the peptoid monomer in DMF before microwaving for 30 s total (6 × 5 s at 100% power; venting and rocking for 30 s in between intervals) before being washed with DMF (5 × 5 ml). This procedure was repeated for each monomer addition. Traditional Fmoc peptide synthesis was followed for the addition of glycine residues, 5 equivalents of Fmoc-Gly-OH, 5 equivalents of hexafluorophosphate benzotriazole tetramethyl uranium, and 5% N-methylmorophiline were incubated for 10 min before being added to the resin and rocked for 1 h at rt. The resin was washed with DMF (5 × 5 ml) and treated with 20% piperidine in DMF (5 ml for 15 min, twice) to remove the Fmoc-protecting group. To incorporate an NArg residue into a peptoid, a 0.5 M solution of 1,3-diaminopropane in DMF was added to the resin and coupled by microwaving for 30 s in 5-s intervals with shaking in between. The diamine solution was removed, and the resin was rinsed with DMF (5 × 5 ml). A Dde protecting group was installed on the primary amine by treating the resin with 10 equivalents of 2-acetyldimedone in 1 ml of DMF. The resin was rocked in the Dde solution for 4 h at 25 °C. The Dde solution was removed, and the resin rinsed with DMF. After Dde installation, the remaining peptoid residues were coupled using previously described methods.

For synthesizing compound P2 (unacetylated version), the N terminus of the completed peptoid was protected with Boc anhydride (di-tert-butyl decarbonate). This was done by treating the resin with 10 equivalents of Boc anhydride and 5% NMM in DMF. The Dde group was removed using a 2% hydrazine solution (dissolved in DMF), rocking for 30 min at 25 °C. The hydrazine solution was removed, and this step was repeated, followed by rinsing with DMF. After Dde removal, a guanidinium can be added to create the NArg residue or ethylchloroacetamidate hydrochloride (*i.e.*, chloroacetamidine warhead) can be coupled. To add the guanidinium, 6 equivalents of 1-aminopyrazole hydrochloride and N,N-diisopropylethylamine was dissolved in anhydrous DMF and added to the resin. The resin was rocked in this solution for 8 h at 25 °C. To add the chloroacetamidine warhead, a solution of 6 equivalents of ethylchloroacetamidate hydrochloride and 6 equivalents of N,N-diisopropylethylamine in anhydrous DMF was added to the resin and allowed to react for 8 h at 25 °C. The solution was removed, and the resin was rinsed with DMF after each step.

Acetylation of the N terminus was accomplished by treating the resin with an acetylating solution (1:1 triethylamine:acetic anhydride in 1:1 DMF:DCM) for 1 h at rt with rocking before washing with a 1:1 mix of DMF:DCM (5 × 5 ml). Note: acetylation was completed prior to installation of the warhead or guanidium groups.

Finally, the resin was treated with 95% TFA, 2.5% triisopropylsilane, and 2.5% water for 1 h to cleave the peptoid from the resin. The TFA was evaporated and the peptoid was precipitated with cold diethyl ether before being redissolved in water and purified by reverse-phase HPLC on a Vydac Protein and Peptide column. The mass of the peptoids were confirmed by ESI mass spectrometry (Table S1).

### IC_50_ assay

The IC_50_ value for the peptoid inhibitors were measured using the MTase-Glo methyltransferase assay kit (Promega). The peptoid was incubated in assay buffer (20 mM Tris-HCl pH 8.0, 50 mM NaCl, 1 mM EDTA, 3 mM MgCl_2_, 0.1 mg/ml BSA, 1 mM DTT) and substrate (0–1000 μM). After a preincubation of 10 min, 200 nM PRMT1 or PRMT5 was added to the assay before adding in the substrate (AcH4-21) at 225 μM. After 15 min, the reaction was quenched with 0.5% TFA (0.1% final concentration), vortexed, and incubated for an additional 10 min. To detect product formation, the MTase-Glo reagent was added for 30 min at rt, followed by addition of the MTase-Glo detection solution for an additional 30 min. Finally, the luminescence signal was measured using a BioTek Synergy two multi-mode microplate reader. The values were converted to product concentration using a standard curve of SAH (0–5 μM). The IC_50_ values were determined using Equation 1 and fit to a curve using GraFit 7.03.

(1) $$\text{Fractional}\;\text{activity}\;\text{of}\;\text{PRMT}=1/\left(1+\left(\left[\text{I}\right]/{\text{IC}}_{50}\right)\right)$$

IC_50_ is the concentration of inhibitor resulting in 50% PRMT activity, and [I] is the inhibitor concentration.

### Cell culture and conditions

MDA-MB-468 breast carcinoma cells (ATCC; HTB132), HCT116 +/+ p53 colon carcinoma cells were grown in DMEM supplemented in 5% FBS. HepaRG terminally differentiated liver cell line (ThermoScientific; HPRGC10) and HepaRG-Fucci cells were grown in Williams media supplemented with Glutamax and 5% FBS and normal HMECs (ATCC; PCS-600-010) were grown in mammary epithelial basal medium (ATCC; PCS-600-030) supplemented with mammary epithelial cell growth kit (ATCC; PCS-600-040). Cells were recovered with 0.25% trypsin-EDTA, plated on cell culture treated plates, and/or coverslips according to the experimental design and incubated overnight in 37 °C humidified CO_2_ incubator kept at 5% CO_2_ (800WJ; ThermoScientific). Cells were washed twice with sterile PBS before switching to serum-free treatment media along with varying concentrations of the PRMT inhibitor compound (0–40 μM).

### Morphology

Cells were grown on cover slips, stained using Diff-Quick kit, and observed for markers of apoptosis and stress which included a stalled, multinucleated, apoptotic, and normal morphology. In addition, the cell body and nuclear diameter, cell body areas, and presence of vacuoles were noted using microscopy and Image J image analysis of three representative sections on each coverslip.

### Digital image analysis

Digital images of coverslips were obtained under 20× and 40× objective lens magnification. Image J software was used to count cells and nuclei. In addition, software was calibrated using a stage micrometer image and then subsequently used to determine cell body & nuclear diameters and individual cell areas.

### Crystal violet cytotoxicity assay

Cell viability and growth was assessed using the crystal violet assay as described by Oliver *et al.* (50). Briefly, cells from various control and pretreated conditions were seeded in 96-well plates in triplicate at a density of 5 × 10^3^ and grown for 72 h in normal cell culture media typically used for each cell line. Cells were fixed at each time point with 100% methanol and stained with 1% crystal violet in 0.01 M borate buffer (pH 8.9). Absorbance of dye eluted in 1:1 (v/v) ethanol: 0.01 M HCl was checked at 650 nm by microplate reader. The cell growth rate was calculated by the following formula: Cell growth rate (%) = (A650 at n hour/A650 0 h). Each condition was tested in triplicate and repeated three independent times.

### Trypan blue assay

Cell viability of HepaRG immortalized cells and MDA468 cells were assessed using trypan blue cell viability assay. Cells were grown in small plates, treated with varying concentrations of the PRMT inhibitor compound (0–20 μM) for 24, 48, and 72 h. At each time point, media was aspirated, and cells were washed with PBS. A volume of 150 ml of 0.25% trypsin-EDTA was added to each plate to detach the cells. Trypsin was then neutralized by 350 ml of DMEM supplemented in 5% FBS. A 100 ml sample of the cell suspension was mixed with 100 ml of 4% trypan blue solution in PBS. Cell viability was observed by counting the blue *versus* white cells within 30 min of treatment using a hemocytometer. The percent of blue and white cells was calculated for each treatment.

### Soft agar colony formation assay

Tumorigenicity of MDA-MB-468 and HCT116 +/+ colon carcinoma was tested using colony formation assay in triplicate. 2% agar mixed with 20% FBS DMEM to create a 1% agar/10× media mixture. A volume of 0.75 ml of the mixture was added into each well of a 12 well plate and allowed to solidify. 0.75 ml of 0.6% agar and media mixture with desired concentration of UnAcH416 was used for the top layer mixed with 2500 cells/well. Plates were incubated in a 37 °C humidified CO_2_ incubator kept at 5% CO_2_ (800WJ; Thermo Scientific) for 2 weeks, with biweekly treatments of compound P2 in 50 μl media/well. Colonies were treated with MTT dye and allowed to dry before imaging and analysis. Colonies were photographed using a stereomicroscope and counted using Image J analysis. The entire experiment was repeated twice.

### Protein extraction and quantification

Cell lysates of cultured cells were prepared using either the RIPA or 8 M urea:4% SDS containing buffer supplemented with proteinase and phosphatase inhibitors. The samples were sonicated for 5 s on ice and centrifuged at 14,000 rpm for 10 min at 4 °C to remove debris. Samples were kept at −20 °C until use. The proteins were quantified using BCA protein assay against a BSA standard curve as per manufacturer’s protocol (cat# 23225, Thermo Scientific).

### Caspase-3 activity assay

Caspase-3 activity assay was performed on extracted protein lysates using colorimetric caspase-3 activity assay (K106; BioVision) as per manufacturer’s protocol. Briefly, 50 μl of extracted protein was plated in a UV compatible 96-well plate. DEVD-pNA substrate in prepared 1× buffer was added to each sample. The plate was shaken for 10 min and incubated at 37 °C in Bio-Rad plate reader and read at 405 nm every 10 min for 2 h. Raw caspase-3 activity data were blank corrected and converted to relative caspase-3 activity compared to untreated control cells based on relative protein amount loaded in the assay.

### Western blot analysis

Expression of proteins was detected by Western blot assay as described previously. Briefly, cell extracts and tumor lysates were separated by SDS-PAGE followed by transfer to nitrocellulose membranes. Membranes were blocked with 5% milk in TBS-T (TBS containing Tween 20) for 30 min to 1 h and incubated in primary antibody (Santa Cruz) overnight at 4 °C followed by incubation in peroxidase-conjugated goat anti-rabbit IgG (1:500, Santa Cruz) for 1 h. Proteins were visualized by autoradiography, and films were scanned. GAPDH was used as the loading control for Western blotting with total liver protein extract. Ponceau staining of each protein column was also quantitated as an additional loading control.

### Statistical analysis

Experimental data were expressed as average ± standard error. Significance for comparisons of two and multiple independent variables were calculated using Student’s *t* test and two-way ANOVA (PRISM software; GraphPad), respectively. A *p*-value < 0.05 (*p* < 0.025 & 0.0166 for 2- and 3-comparison experiments, respectively) was considered statistically significant.

## Data availability

All data are contained within the manuscript.

## Supporting information

This article contains supporting information.

## Conflict of interest

The authors declare that they have no conflicts of interest with the contents of this article.
