# Supplementary material for: A peptoid-based inhibitor of protein arginine methyltransferase 1 (PRMT1) induces apoptosis and autophagy in cancer cells
Source: J Biol Chem. 2022 Jun 25;298(8):102205. doi: 10.1016/j.jbc.2022.102205 (PMC9307946; doi:10.1016/j.jbc.2022.102205)

Supporting Information

A peptoid-based inhibitor of Protein Arginine Methyltransferase 1 (PRMT1) induces apoptosis and autophagy in cancer cells

Mollie A. Brekker[1], Tala Sartawi[2], Tina Sawatzky[1], Corey P. Causey[1], Fatima Rehman[2], and Bryan Knuckley[1]

[1]Department of Chemistry, University of North Florida, Jacksonville, FL, U.S.A.
[2]Department of Biology, University of North Florida, Jacksonville, FL, U.S.A.

**Table S1.** Mass of Synthesized Peptoids		
**Peptoid/Peptide**	**Experimental Mass**	**Observed Mass**
Compound P2	1459.8	1460.8
Compound P2A	1501.8	1524.6 ($^{M+Na+}$)
Compound P2 - Peptide	1445.8	1446.7
Compound P2A - Peptide	1487.8	1488.7

Supplemental Table 1. List of synthesized peptides and peptoids with mass values. The full spectra can be found below.

A.

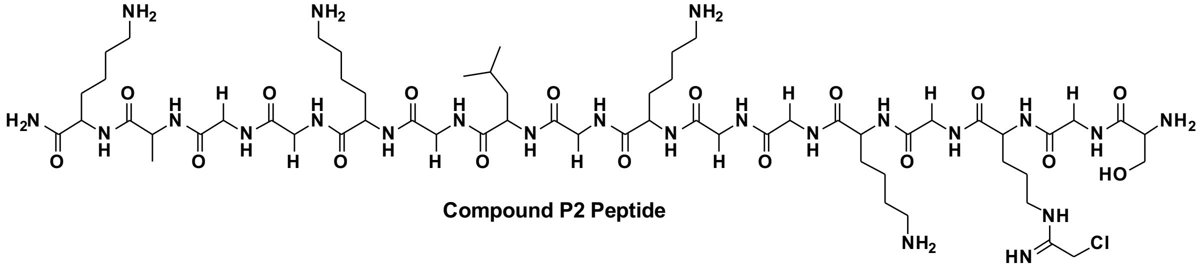

B.

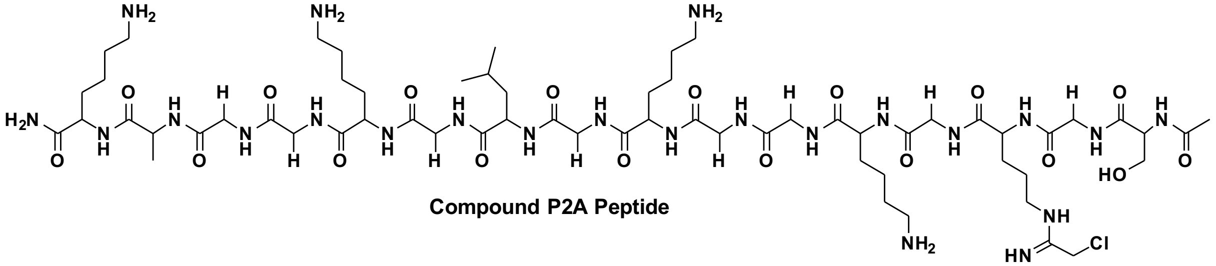

Supplemental Figure 1. Structures of the peptide version of (A) Compound P2 and (B) Compound P2A.

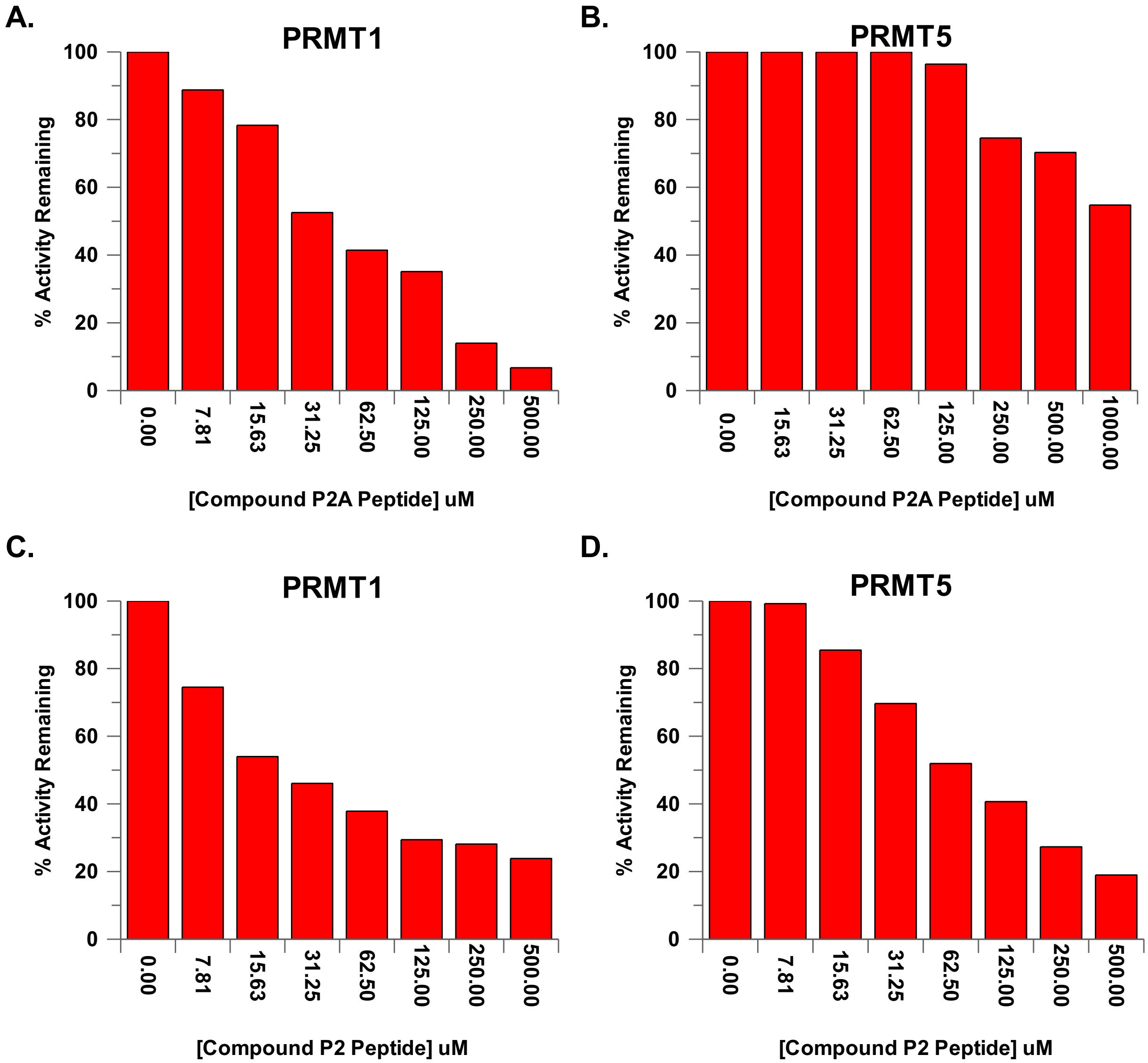

Supplemental Figure 2. IC_{50} plots of Compound P2A peptide with (A) PRMT1 and (B) PRMT5. IC_{50} plots of the Compound P2 peptide with (C) PRMT1 and (D) PRMT5.

Compound P2

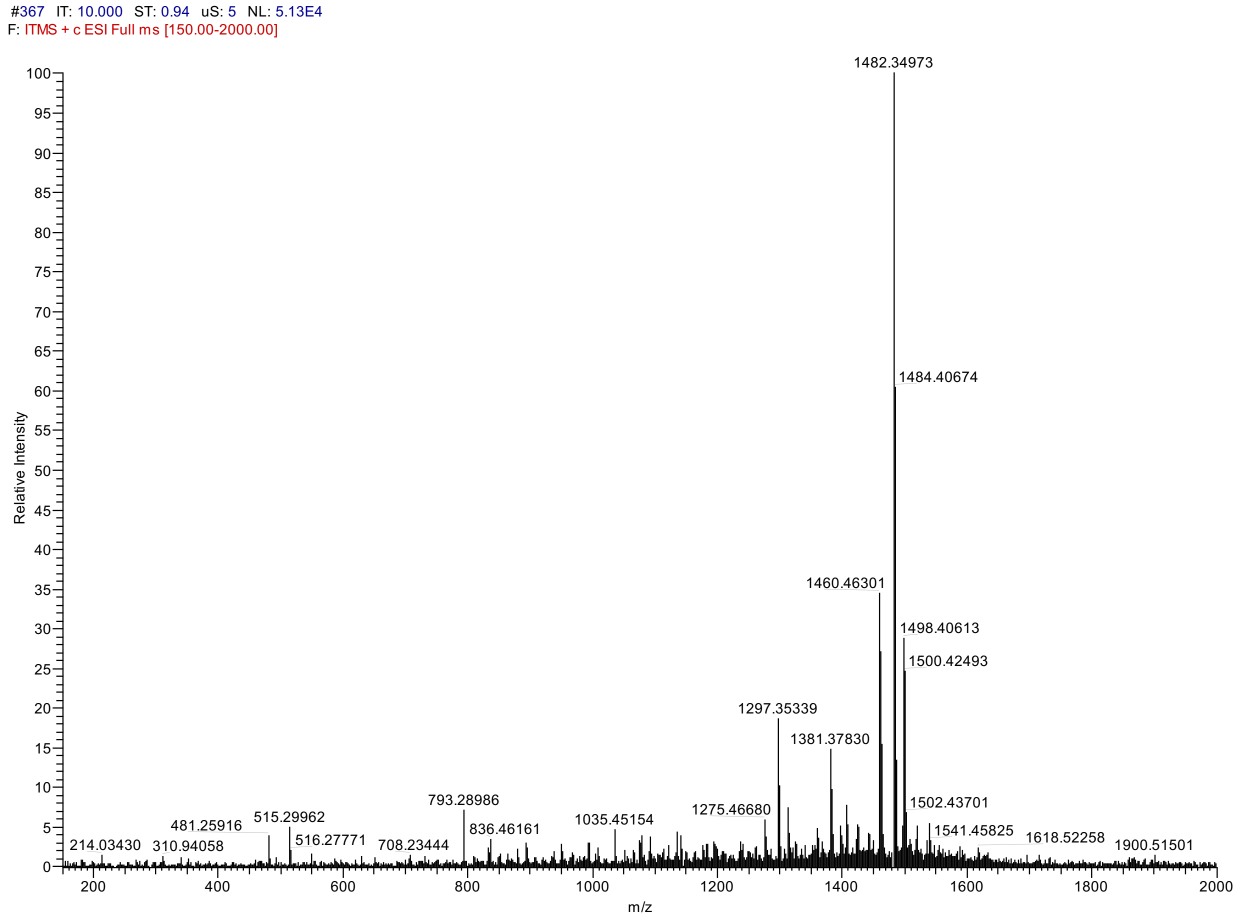
#367 IT: 10.000 ST: 0.94 uS: 5 NL: 5.13E4
F: ITMS + c ESI Full ms [150.00-2000.00]
Relative Intensity
m/z
1482.34973
1484.40674
1460.46301
1498.40613
1500.42493
1297.35339
1381.37830
1275.46680
1502.43701
1541.45825
1618.52258
1900.51501
1035.45154
793.28986
836.46161
708.23444
515.29962
516.27771
481.25916
310.94058
214.03430

Compound P2A

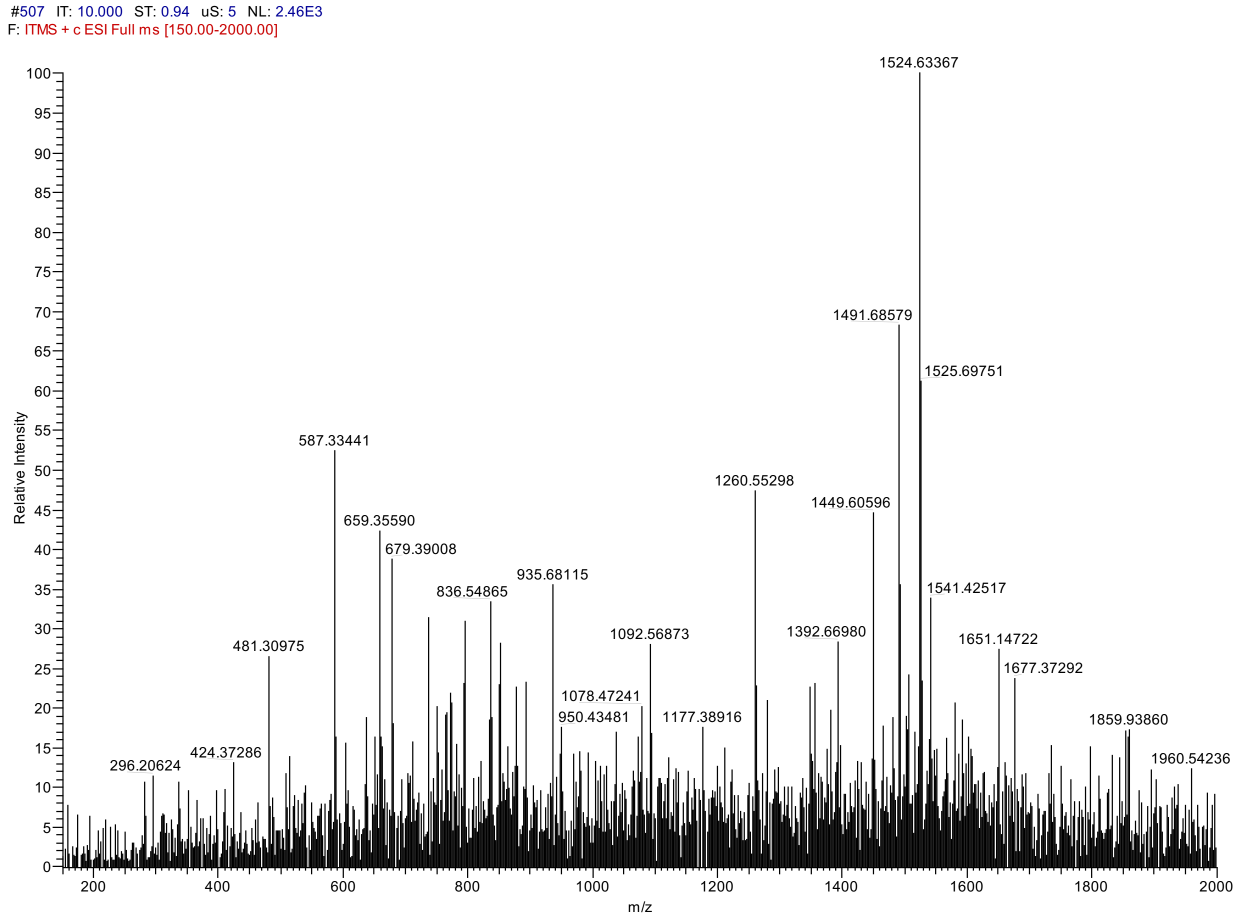

Compound P2 Peptide Version

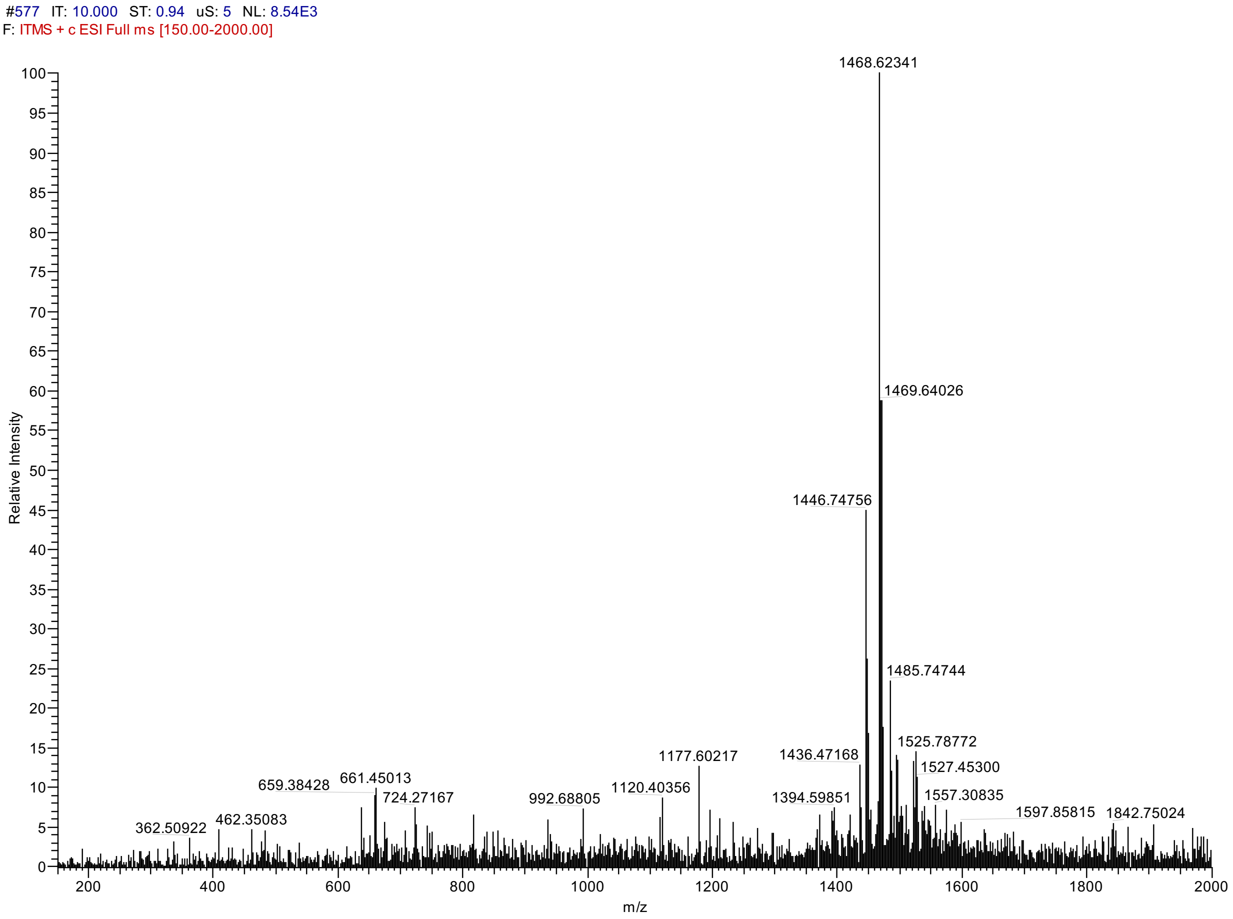

Compound P2A Peptide Version

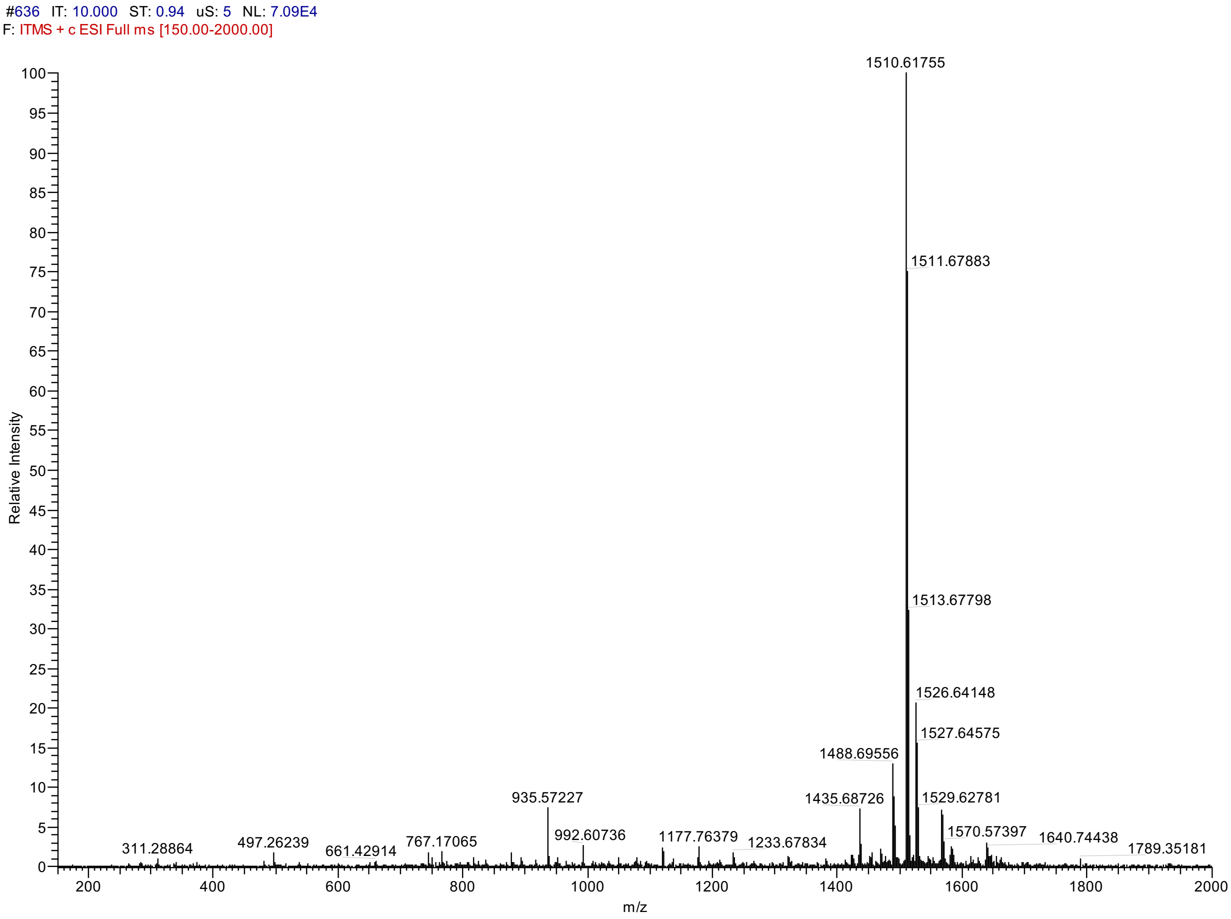

Supplement: Supplemental Table S1, Figures S1 and S2 [file mmc1.pdf]
